# Access to gender-affirming hormonal therapy in Russia: perspectives of trans people and endocrinologists

**DOI:** 10.1080/26895269.2023.2272145

**Published:** 2023-10-23

**Authors:** Yana Kirey-Sitnikova

**Affiliations:** BMC Chemistry, Uppsala University, Uppsala, Sweden

**Keywords:** Gender dysphoria, hormone replacement therapy, Russia, self-medication, transgender

## Abstract

**Purpose:**

Worldwide, trans people are known to take hormones without prescription or take higher or lower doses than prescribed. The study’s goal was examination of the social context behind provision and receiving of gender-affirming hormonal therapy (GAHT) in Russia, as well as collection of opinions on the organization of healthcare.

**Methods:**

Semi-structured interviews with 20 trans individuals and 6 endocrinologists.

**Results:**

Trans respondents started GAHT between 2004 and 2022. Over that period, the number of trans-competent endocrinologists increased and it became easier to receive the diagnosis F64.0 “transsexualism” (ICD-10), which qualifies one to obtain GAHT. However, the majority still took hormones without supervision. The reasons for self-medication included: the lack of the diagnosis, high cost of appointments and tests, age under 18, and self-perceived understanding of principles of GAHT. Most endocrinologists learnt about GAHT after being approached by a trans patient. They received information from advanced training courses and academic literature. The specialists had diverse opinions on the ideal policy of providing GAHT to trans people, but all claimed that in Russian political realities they found the diagnosis “transsexualism” a necessary prerequisite for initiating GAHT to safeguard both doctors and patients.

**Conclusions:**

The lack of strict law enforcement in the pharmacological sphere in Russia enabled many trans people to initiate GAHT without supervision. Trans-competent endocrinologists were available for those who needed them. Under current political circumstances it is unlikely that the informed consent model (prescription without a psychiatric diagnosis) can be implemented in Russia.

## Introduction

Medical care for trans patients has been provided in the Soviet Union since late 1960s. Psychiatric assessment was performed by Dr. Aron Belkin in Moscow, followed by gender-affirming hormone therapy (GAHT) prescribed by Dr. Irina Golubeva. Later, trans healthcare spread to other parts of the country, including Rostov-on-Don and St. Petersburg (Kirey-Sitnikova, 2022). In 1991, the first Soviet clinical practice guidelines “Transsexualism (Methodological Recommendations on the Change of Sex)” were approved by the Ministry of Health (Ministry of Health of the USSR, 1991). These guidelines recommended prescription of cyproterone acetate to both trans women and trans men followed by ethinylestradiol for trans women and testosterone cypionate for trans men. In post-Soviet Russia, development of medical care for trans patients continued. In 2006, Svetlana Kalinchenko published the first book fully devoted to endocrinological care for trans patients (Kalinchenko, 2006). In the past decade, access to GAHT was further improved by the emergence of a young generation of doctors who received advanced training from trans activists (Kirey-Sitnikova, 2022). Simultaneously, trans people are more frequently becoming targets of state repressions. In November 2022, the State Duma adopted a ban on “propaganda of the change of sex,” leading to blocking of several websites providing information on trans issues, including medical care. In July 2023, another law prohibited legal gender recognition and gender-affirming care (including GAHT) to those who had not legally recognized their gender before.

The data for this project was collected in September-October 2022, before adoption of these laws. At that time, the official process of gender transition in Russia began with an evaluation by a medical commission consisting of a psychiatrist, sexologist, and medical psychologist. The commission (which could be established in any clinic, public or private) issued a “certificate on the change of sex” that was a prerequisite for legal gender recognition, prescription of GAHT and undergoing surgeries. Since the field was not clearly regulated, some endocrinologists prescribed GAHT based on the diagnosis F64.0 “transsexualism” (ICD-10)^1^ issued by one psychiatrist. Psychiatric assessments and consultations with endocrinologists were provided in public and private clinics. Since the quality of care in public hospitals is generally low, trans people who had enough money chose private doctors (Kirey-Sitnikova, 2022). Hormones could be bought without a prescription in pharmacies or illegally on special websites. Thus, from 53.6% to 59.4% of Russian trans people initiated GAHT without a prescription, according to two large surveys (Chumakov et al., 2022; Makarova et al., 2021).

This paper seeks to explore experiences of trans people and endocrinologists in the sphere of GAHT. Trans people were specifically questioned on the reasons they chose to take GAHT under supervision or without it, what were their experiences with endocrinologists, how and where they managed to buy medications, and what they thought about the informed consent model. As for endocrinologists, their opinions on self-medication and organization of healthcare were requested.

## Methods

Interview questions were developed based on the author’s personal experience as a trans individual and activist working with trans issues in Russia for 13 years, as well as an analysis of the literature. In addition, the author holds a Master’s degree in Public Health. This dual positionality enabled the author good access to and mutual understanding with both trans respondents and doctors.

Trans participants were recruited *via* social networks and asked to fill a Google Form that included the description of the study and fields such as gender identity, age, place of residence, the year GAHT was initiated, whether GAHT was taken under endocrinological supervision, and contacts for further communication. Trans people of all ages with an experience of taking GATH in Russia were eligible. A total of 49 responses were recorded. Out of them, 25 were selected to ensure diversity of gender identities, geography, and experiences. Of these 25 contacted, 18 gave interviews. In addition, three individuals contacted the researcher directly without filling the form. Thus, 21 interviews were taken in total. In the beginning, the researcher briefly introduced the interviewees to the study, assured confidentiality, and asked whether they agreed to participate. The language of interviews was Russian. While most interviews were oral and conducted online, 3 participants asked for a written interview. Trans participants were compensated with 300 rubles (∼5 USD) for their time.

Likewise, a Google Form for endocrinologists was created, it contained the fields: name, place of providing care, and contacts. Since only two respondents were recruited using this form, endocrinologists from the lists of trans friendly doctors maintained by NGOs were contacted directly. One doctor was recommended by a trans respondent. Altogether, 7 endocrinologists were interviewed. No financial incentives were offered to endocrinologists.

Upon analysis of the data, it turned out that one “trans respondent” and one “endocrinologist” were probably the same person, as they used accounts registered on the same phone number. It is not clear why this false respondent decided to participate twice. These two interviews were excluded, reducing the number of respondents to 20 for trans people and 6 for endocrinologists.

The remaining interviews were used for thematic analysis based on the reading of transcripts through the lenses of the author’s personal experience as a trans person and activist. The codes were assigned in the software Taguette. The code book was initially developed based on interview questions and additional codes were added inductively upon going through the transcripts. Later, the codes were merged into broader themes. The quotes were translated into English by the author.

## Results: trans individuals

The 20 participants in the sample had a median age of 28 (range: 15 to 47). There were ten trans men, seven trans women, and three non-binary individuals. The median year of starting GAHT was 2015 (range: 2004 to 2022), one participant received a prescription for GAHT but did not initiate it at the time of the interview. Seven respondents were from Saint-Petersburg, three—from Moscow, others lived in Kaliningrad, Sochi, Smolensk, Vladivostok, Novosibirsk, and undisclosed locations in Murmansk, Leningrad, and Moscow Oblast, as well as Krasnodar Kraj.

### Initiating GAHT

One fourth (5) of the respondents started GAHT almost at the same time they decided to transition. For others it took up to 6 years. The most common reasons for delaying GAHT were being young (and, by consequence, being dependent on parents and having no money of their own) and the need to find more information. For example, a 19-year-old man revealed he had to wait for 6 years: “I fully realized [that I was trans] at 13, I had to wait until I could start at 19. [Why?] Before that, I had thoughts but no capabilities. [Financial capabilities or you were waiting for the diagnosis?] Financial.” One respondent mentioned health concerns (specifically problems with blood vessels and panic attacks) as a reason for hesitation, which led to a delay: “My health is not perfect. I searched in Google, asked people, visited doctors. When I became convinced that HRT^2^ will be beneficial for me, I started” (Non-binary trans masculine, 47).

### Choosing between GAHT under supervision and self-medication

None of the respondents was consistently taking GAHT under supervision of an endocrinologist: nine never visited an endocrinologist, three started GAHT under supervision but later chose self-medication, four started with self-medication but later approached an endocrinologist, while three alternated self-prescribed and doctor-prescribed therapy throughout their lives. Four main reasons for not consulting endocrinologists emerged from the data: perceived lack of need, lack of professionalism on behalf of endocrinologists, barriers in access, and anticipated mistreatment.

#### Perceived lack of need

A trans woman (26) reported that there was no perceived necessity for approaching endocrinologists in the community: “Out of my acquaintances, no one went [to an endocrinologist], TS [transsexuals] I knew. Everyone uses HRT but no one went.” This respondent further explained that being on hormones might help her receive the diagnosis “transsexualism.” A 43-year-old trans woman had an in-depth understanding of GAHT because of her background in medicinal chemistry: “The reason is the lack of need for me personally: I have enough knowledge, medications are available without a prescription. The schemes are chosen based on test results.”

#### Lack of competence

Some participants who had experience with endocrinologists complained about their lack of professionalism, which led them to choose self-treatment. One trans masculine respondent (47) reported: “I visited [endocrinologists] previously, then I lost trust and did not know where to find a competent one.” One doctor prescribed him medications that caused a side effect, in another place the doctor refused to help him. In the end, he chose to continue the scheme prescribed by the last competent doctor on his own. A trans woman (21) had a similar experience: one doctor’s prescription caused a side effect, she found a better one, but that doctor left Russia for political reasons. Over time she became more knowledgeable and felt she could continue GAHT on her own.

#### Barriers in access

Respondents reported varying barriers, including cost, young age, legal restrictions, and the diagnosis. Trans friendly endocrinologists are usually available in private clinics and demand tests that patients need to cover out of pocket. Thus, financial barriers could lead to self-treatment:

In the end of April, I received the permission, the diagnosis F64.0. And I decided to start everything in an official way: buy medications in a pharmacy and make an appointment with an endocrinologist. But the first thing that stopped me was a long list of tests for the endocrinologist from “Centre-T” that cost more than 10 thousands rubles for an initial appointment (Trans man, 19).

Minors cannot receive GAHT officially in Russia, that is why some of them start transition without supervision. However, in one case, an endocrinologist provided a consultation to a trans minor in an unofficial way:

She provided me with general knowledge, warned about risks. She said that officially, of course, she cannot recommend, but in general it works so and so. As I understand it, I was not the first person who approached her without the diagnosis, so I think she understood that I will start hormone therapy anyway, the question is how correct it will be (Trans man, 28).

One trans man (43) chose to buy testosterone illegally after official purchase in pharmacies became more strictly regulated: “Previously recipes were simple, without a special form. But then they started requiring a special form, so [the endocrinologist] made the last prescription for a large amount. I bought for several years in advance, then moved to unofficial.”

Not having the diagnosis “transsexualism” qualifying one for official prescription was another barrier since receiving the diagnosis could take considerable amount of time (sometimes up to two years) and money:

In May, when I was 18, I had a commission [psychiatric assessment], and just a little earlier, before spring, in the end of winter, I smeared myself with hormones (Androgel®), because I thought I won’t pass the commission soon, because in a free polyclinic [public hospital], if you know, in the dispensary, you need to be observed for two years, so receiving hormones would be postponed for two years (Trans man, 20).

#### Anticipated mistreatment

Only one trans woman (31) reported the fear of transphobia as a reason for avoiding endocrinologists.

The main reason for visiting endocrinologists were concerns about possible health risks: “I am a bit worried for my health … I heard all these horror stories about varicose, cancer, etc. I have heredity in this regard, so I trust the endocrinologist” (Trans woman, 34). Another concern was illegality of buying hormones without a prescription: “Because I knew that they are prescription medications. I understood that them selling me without a prescription is not right and not quite legal. This can stop at any moment” (Trans man, 35). One trans woman (43) was not satisfied with the results of GAHT and approached an endocrinologist to understand the reasons: “I had to figure out. [Antiandrogens] definitely do not give an effect, because all the changes [in appearance] result only from cosmetological procedures that I do. If I stop, I will lose feminine pass after a while.”

### Experiences with endocrinologists

Trans people who started GAHT in the 2000s reported the lack of competence on behalf of endocrinologists, even though they were eager to learn. A trans man (35) who started GAHT in 2008 recalled:

First, I went through [doctors] literally by guesswork, and not all were friendly. Sometimes I had to give long explanations what and how, show the paper, but in the end I found in a private clinic… Great endocrinologist. First, she was not aware, but after I visited her, she asked colleagues from Moscow who help trans people, she read the literature.

In the 2000s and early 2010s, endocrinologists could prescribe hormones without an official diagnosis. A trans man (43) who started GAHT in 2009 recalled that a paper from a psychiatrist stating that the person was being evaluated for transsexualism was enough:

Then the situation around transgenderism was completely different. Fewer transitioned, there were more fears. There were criteria, the ideas about transsexualism within the community were more rigid, medicalized … But I went to [name of an endocrinologist], I had only a paper from [name of a psychiatrist] stating that I am being evaluated for something… I do not remember. But I did not have a medical certificate. I came, I do not even remember whether I showed this paper or not. But I had a good pass. I came and said: “I need hormone therapy.” She: “Yes, I see.”

Over the years, two trends could be observed: endocrinologists started consistently requiring the diagnosis “transsexualism” (although no change in legal framework occurred; for the perspective of doctors see next part) and more endocrinologists became trans competent thanks to efforts of trans activists. One trans NGO asked volunteers to visit endocrinologists and distribute the literature:

When “T-Action” had a campaign on engaging doctors and distributing the [WPATH] Standards [of Care], books “Trans people visiting a doctor,” everyone visited endocrinologists in their polyclinics and handed out translated Standards of Care, 7th version. That is how I received a prescription (Trans man, 28).

The doctors who got interested were later invited to participate in an advance learning course that was being organized by trans activists in cooperation with the state-run Almazov National Medical Research Center. Lists of trans friendly and competent doctors who undertook this training were later put on websites of trans NGOs, so that trans people could contact them.

Although endocrinologists became better aware of the needs of trans people, patients were not fully satisfied: “The main problem with [name] is that she has many patients … And unfortunately, her approach is very assembly-line … She does not go into details. She has template answers, she speaks with patterns and linguistic clichés” (Trans woman, 21). Such an indiscriminate, superficial approach resulted in side effects for two participants. In one case, a trans woman who had had orchiectomy continued to receive medications with an antiandrogen effect that led to very low levels of testosterone. Instead of choosing other drugs, the endocrinologist added a gel form of testosterone: “The scheme was standard, non-individualized, and when individualizing became necessary because of external circumstances, she suggested complexification, adding medications, instead of balancing the scheme to make it less expensive, less resource-intensive for the organism” (Trans woman, 21). This was one of the reasons this participant later chose self-treatment. A similar situation happened with another participant: “First I liked her. But then it turned out I had various side effects… after several years. But she never paid attention, although in the tests it was seen, she did not react at all. Thus, I stopped visiting her” (Non-binary trans masculine, 47).

Endocrinologists who had no training in trans healthcare could simply refuse to help: “Once they did not give me a prescription in a private [clinic], despite all my certificates. They were frightened, decided… got frightened, refused, returned money” (Non-binary trans masculine, 47). The situation in state-run clinics is even worse: “I will never set foot in this polyclinic, because, first of all, without my consent they tell all the doctors who I am, and second, getting a prescription is very problematic, because every visit I have to prove who I am” (Trans man, 35).

### Choosing medications

Whether the participants consulted an endocrinologist or engaged in self-treatment, GAHT typically started with a standard scheme, which was corrected according to test results and satisfaction with changes in physical appearance. One participant described how it works: “You make an injection, in the middle of the cycle you go to a polyclinic or a private clinic to take a blood test. You bring back the test results, she looks at them and says whether everything is normal or not” (Trans man, 20). Those who chose self-treatment received information from the internet or other trans people. Often choosing a scheme involved experimentation:

Based on recommendations, then on personal experience. I look at how, for example, the medication affects my face, when it starts retracing… Or I look at test results, and I do not do it that way. I experiment, in short. [Can you really see from your face how it changes?] Yes, it becomes… Now it is this rounded. It becomes angular when I make an injections less frequently, estradiol injections (Trans woman, 26).

Experimentation could lead to undesirable effects:

I take tests at certain days and see. If they are within the range that I want to see, then I do not change the scheme. Once … I experimented with the scheme … My schedule was 1 ml [of Sustanon®] once in 18 days, that is 250 mg. And I tested what will be if I reduce the time twice and reduce the dose twice … So, I moved to 0.5 ml in 9 days… Approximately one month later, I noticed manifestations that looked like an ovulatory cycle, I mean bloody discharge … I returned to my previous schedule, but to my surprise the situation did not change. It changed after I drastically reduced the time. I mean not 18 but approximately 14 days (Trans man, 28).

Moving from one medication to another could be motivated by financial reasons or unavailability of certain drugs (for access problems see the next section):

First I moved from Proginova® to Divigel®, because Proginova® became very expensive. Then from Divigel® to Femoston®, because there was not enough estradiol in Divigel® for me. And then I took Femoston® + Chloe®, it was since New Year until June. Then there was Synestrol® and later, around August, I received the first batch of injections. Transition from one medication to another was related to the lack of money, so I was moving to a cheaper option, or the amount of hormones was not enough (Trans woman, 29).

### Buying hormones

Feminizing hormones and testosterone blockers are generally available in Russian pharmacies without a prescription. A prescription is officially required, but pharmacists do not check for it, although it depends on the place: “Sometimes they ask [for a prescription]. Most often they do not ask, but I have it with me just in case” (Trans woman, 26). Even if they ask for a prescription, it is always possible to go to another pharmacy where the practices are different. Thus, none of the trans women interviewed ever had problems buying hormones without a prescription. Despite availability of feminizing medications in pharmacies, some trans women chose to buy them abroad stating that it saved them money: “I order through a person. Estradiol from India through another person. [Why from India?] If I order the Japanese, original, it will… I do not even know whether I can afford that money” (Trans woman, 26).

Androgel® is the only testosterone-containing medication, which can be bought without a prescription in Russian pharmacies (a prescription is officially required but rarely checked for): “First [I started] unofficially with Androgel® that can be bought rather easily and without fake prescriptions. You just go to a pharmacy” (Trans man, 20). Other testosterone-containing medications had been sold in a similar vein before control was tightened in 2016:

Before 2015, it was without a prescription, maybe before 2016. Testosterone was a provisionally prescription drug, and you could… A prescription was necessary, but everyone knew, almost in all pharmacies in Saint-Petersburg… You come, buy 8 packs of Omnadren®… They ask: “Why so many?” [I:] “I need it.” And they sold without a prescription. But later they tightened control over the turnover, [testosterone became] a drug under special control: a lockbox is required, a special prescription signed with the doctor’s blood [jokes], three seals. The control became similar to the control over the turnover of narcotic drugs (Trans man, 43).

Not being able to buy testosterone without a prescription was probably the reason why trans men were more likely to approach endocrinologists than trans women. However, even having a prescription did not guarantee that buying testosterone would be easy:

[The doctor] suffers every time because every time he writes a prescription some pharmacy says that a comma is in the wrong place. And I go in three rounds: either find another pharmacy, or we rewrite the prescription … Recently he started giving a prescription for three ampules, and in some pharmacies they give, in others they do not. In some [pharmacies] they give one [ampule] and take the prescription, in some they give three [ampules], if they have. So, there are many difficulties. [Can the doctor give several prescriptions at once?] In the clinic, it is not encouraged (Non-binary trans masculine, 47).

Furthermore, geographic inequalities exist, as it is not possible to buy certain medications in smaller towns and villages:

I live in a small town in the Far North, in Murmansk Oblast, and there neither Omnadren®, nor Nebido® were imported. And it was not possible to order them [online] through pharmacies because they go under prescription … Constantly going to the regional center is very expensive, it takes 5 hours by car to buy one drug (Trans man, 19).

Faced with barriers to receiving testosterone in an official way, some trans masculine people chose to buy it illegally on websites whose main clients are cisgender bodybuilders. Those medications come from a variety of foreign sources: “As I understand, they order directly from foreign suppliers. I do not ask, do not interfere. I do not tell anyone that I am on self-prescribed HRT, I do not share contacts of this person to avoid implicating him accidentally” (Non-binary trans man, 28). The drugs are probably produced illegally, yet no one complained about their quality:

Based on test results, I do not see any difference. To check other [aspects of] quality, I would need a quality control laboratory. There is no difference in external appearance of the oily solution, in fluidity. As for the effect… the effect is present. It does not cause side effects such as allergy, but I do not have allergy in general. Thus, it suits me well (Trans man, 28).

The so called “special military operation” inaugurated by Russia against Ukraine led to Western sanctions and, by consequence, supply disruptions for many drugs imported from Europe, including hormones:

During the first few months after 24 February 2022, there were disruptions in supply of HRT and a sharp rise in prices, around March-May. In March, I had to reduce the dosage of estrogens for a couple of months. Now everything returned to the situation before 24 February 2022, but the fear that medications will disappear remains (Trans woman, 31).

The lack of prescribed medications stopped one participant from initiating GAHT: “In the pharmacies, there is no medication that I was prescribed. In the beginning of July, I went to an endocrinologist, I was prescribed Divigel® and Finasteride®. As you probably know, there is no Divigel® now” (Trans woman, 34). The informants who bought online also reported some changes:

The price grew a bit, and the assortment narrowed. Previously there was a choice between 4-5 producing countries, now it went down almost to one country … Previously I mainly bought Moldovan. China, India, and… I forgot what else. And Dutch also. Now the only left are India and China (Non-binary trans man, 28).

Others reported problems during the COVID-19 pandemics: “During COVID-19, it was a nightmare, because all clinics closed down, you could not visit a doctor, could not receive a prescription” (Non-binary trans man, 47). Despite these disruptions, all the respondents managed to continue GAHT without major interruptions. Only one trans woman had to stop taking hormones for 4 months to undergo vaginoplasty, as requested by the surgeon.

### Organization of healthcare

Half of the participants (10) supported the informed consent model, meaning that psychiatric assessment is not required to prescribe GAHT (Schulz, 2018): “I am in favor of individuals independently making decisions about their HRT, their body, identity. So, I am not sure that diagnostics is necessary at the legal level. As they say, my body is my choice” (Non-binary trans man, 28). Several interviewees did not know the meaning of the phrase “informed consent model” but upon explanation stated that it might be a good solution. Fear of regret was the main reason why other seven respondents preferred prescription of GAHT upon diagnostics. Yet, sometimes their logic was skewed, as they cited cases of detransition following a psychiatric evaluation, meaning that diagnostics had no preventative effect on regret:

If without the diagnosis, then people whose diagnosis is not transsexualism… can make a great mistake and blame doctors … I have a friend who thought that she was a trans guy, but then… The doctor gave her the diagnosis. But then she said: “It is not for me, I made a mistake” (Trans man, 19).

Interestingly, diagnostics was supported even by some respondents who initiated GAHT on their own without the diagnosis: “At some point, I neglected these precaution measures but I do not advise anyone to go that way” (Trans woman, 26). Finally, three interviewees were hesitant on the best model of healthcare provision: “Among transphobes, there is a popular rhetoric that doctors do not care and give hormones to whoever wants, and diagnostics protects against such misconceptions … But if we lived in a more accepting and conscious society, I would choose informed consent” (Trans man, 15).

## Results: endocrinologists

Two endocrinologists came from Saint-Petersburg, two from Moscow, one from Perm Oblast, one from Krasnodar Kraj. All six were cisgender women. There are very few endocrinologists working with trans people in Russia, thus, to ensure their anonymity, no further demographic information was collected. In the quotes below, they will be identified as respondents A, B, C, D, E, and F.

### Motivation and obtaining information

Trans healthcare is not included in the standard curriculum in Russian medical institutions. All the endocrinologists interviewed told that they became interested in the topic after being approached by a trans patient: “A patient approached me. And I just realized that I was asked for help but I have no idea how to help. That is how I got interested” (Resp. C). The doctor who started working with trans patients in 2009 complained that there was not enough information in Russian: “I read textbooks. But in reality there was no information … But how to manage him… at that moment her… in terms of estrogens I did not know” (Resp. A). A book by Svetlana Kalinchenko published in 2006 (Kalinchenko, 2006) used to be the only source in Russian but, according to one respondent, “it is more of a discussion, there are no specific dosages or anything” (Resp. E). WPATH Standards of Care 7 were translated into Russian twice (because of disagreements over terminology among activists) and could be used by endocrinologists who started providing care later. In addition, articles in English could provide guidance for those who knew this language: “English articles. The 7th Standards, of course. At that time they were the 7th, in Russian, in English. From the society I also found… European Society of Endocrinology… on hormone therapy” (Resp. E). Five out of six respondents received advanced training on trans healthcare organized at the initiative of trans activists in the Almazov Center in Saint-Petersburg—the only center offering this kind of training in Russia. They not only received knowledge but also access to patients: “I underwent a training in the Almazov Institute thanks to support from ‘T-Action’ [a trans-led group]. They announced a competition, I participated and received a training. It was in 2018. After that… Probably they have some register of doctors. Many trans people started approaching me” (Resp. A).

### Is working with trans patients difficult?

The endocrinologists felt that providing care to trans patient was not difficult: “In the medical aspect, it is easier to prescribe gender-affirming therapy than archive ideal compensation of diabetes, for example” (Resp. E). Prescribing GAHT to trans men was described as less complicated compared to trans women:

For men, it is not difficult because you encounter testosterone very often, its prescription. And often cisgender men often need a prescription for testosterone therapy. But for girls, women… We are not gynecologists and encounter it less often. And there are many such issues, and in addition, all these therapies related to female hormones, they are rather individual, depending on the person and… whether it is a cisgender woman of a reproductive age, or already in menopause, or a transgender woman (Resp. C).

Most doctors had no difficulty using the patient’s chosen grammatical gender and name. Only one doctor reported some problems at first (she is using “he” because the patient subsequently detransitioned):

First, before I received training, it was confusing, because I had no experience and it was a single patient, plus he was very flamboyant in appearance: then he had a bright horsehair, he chose bright clothes. And this always caused vigorous excitement among nurses and patients in the queue, because it was a municipal polyclinic. Then I had no experience to manage the situation (Resp. A).

### Attitudes to self-medication

The endocrinologists confirmed that trans women more often self-medicated than trans men: “If we talk about girls, then almost everyone. I had three girls and all three initiated therapy before going to a doctor … Boys—no. Boys do it more traditionally” (Resp. A). With improving access to qualified care the prevalence of self-prescription probably went down: “In the beginning, [self-medication] occurred quite often, but now probably there is information, access, all these groups, and all these steps are explained” (Resp. C).

According to one doctor, self-medication can be safe under certain circumstances: “Taking into account the amount of information, if the information comes from trusted sources, I think it is sometimes rather safe” (Resp. A). According to another respondent, it is safe only for individuals with medical knowledge: “If, for example, the person is a doctor, then why not? Will understand. But if the person is far from medicine, I think, control is necessary” (Resp. C). But the general attitude to self-prescription was negative:

Hormonal reconstruction of the organism is happening, all metabolic processes are changing, all tissues… Of course, it is dangerous. There may be some chronic diseases, which can manifest themselves. If the patient had a predisposition to oncology, there was oncology among relatives, it can encourage development. Or a predisposition to thrombosis (Resp. D).

In addition to risks related to cancer and thrombosis, the endocrinologists mentioned the following problems that may arise when wrong schemes of GAHT are used without control: increased levels of prolactin, liver deterioration, heart attack, increased blood pressure, menstrual discharge, skin problems, and fatigue. Although there was no specific question asked, one doctor expressed understanding of the reasons why some patients prefer self-treatment: “I can look at the situation through the eyes of a patient for whom receiving treatment is expensive: taking tests and going to appointments. It can be financially burdensome” (Resp. A).

### Algorithms of treatment

Since no clinical practice guidelines on trans healthcare currently exist in Russia, endocrinologists could choose the best treatment approach on their own. The process generally started with tests:

I recommend to take tests: full blood count, biochemical. If it is feminizing therapy, then abdominal ultrasound. If the person is older than 45, then, of course, ultrasound of the prostate gland or prostate-specific antigen, coagulation test … If it is masculinizing therapy, then pelvic ultrasound, ultrasound of the mammary glands, a smear for oncocytology (Resp. B).

The treatment usually starts with a standardized scheme, which is then individualized: “Common standardized scheme. Separate, of course, for trans masculine persons, for trans feminine. And then they come with test results, I look whether further evaluations are necessary or not. Everything depends on age, on health condition” (Resp. B). The need for an individualized approach for the patients’ different demands was highlighted by almost all respondents:

I have many patients who want partial transition, androgynous appearance, i.e. feminize themselves a bit or, on the contrary, masculinize a bit. Thus, many different requests: someone wants to plan genetic children, someone strongly not … I try first to ask the patient (Resp. B).

### Pressure for providing care

None of the endocrinologists reported persecution on behalf of the police or other state agencies for providing GAHT. They also found understanding among their colleagues: “I always had a very positive experience, and they help me in this regard, and in the clinic also… When we talk about ultrasound, gynecologists… On the contrary, the managers and colleagues are interested” (Resp. C). One respondent used to be head of a private clinic and tailored it to the needs of trans patients:

I used to be a manager for myself for many years. Of course, no. On the contrary, it was easier for me to organize it. I educated the personnel, warned everyone that we will have transgender patients, how they should be addressed, how to treat them ethically—all these ethical aspects we discussed with the personnel. I may say that I built a friendly clinic for TG people (Resp. A).

Parents of trans people of all ages but especially the younger ones were the only individuals who caused trouble:

Sometimes they come with the patients or sometimes even without patients, they come by themselves and tell: “Treat him for me, so that he becomes what he was, or she becomes what she was. Do not support this delusion, bring everything back as it was” (Resp. A).

Parents often wrote complaints to the chief doctor of the clinic or the police. However, none of those complaints caused much trouble: “I tell them that the patient is 18, I have no right to break medical confidentiality. I try to explain that the diagnosis had been officially issued. Then they go file complaints against me. I answer their complaints in writing” (Resp. D).

### Organization of healthcare

The rules of providing GAHT were nowhere clearly defined, so endocrinologists were free to impose whatever requirements they found right for initiation of GAHT. For some, the diagnosis F64.0 from one psychiatrist was enough: “A commission is not necessary, only a conclusion from a psychotherapist or psychiatrist that the doctor issues the diagnosis F64.0 and recommends a consultation with an endocrinologist to start HRT” (Resp. C). Others believed that the “certificate on the change of sex” (issued by a medical commission) should be required. An endocrinologist who had one detransitioner among her patients explained:

Burned on a patient whose life got seriously ruined, who first made a decision, underwent a surgery, changed legal gender, changed all documents, lived like that for several years and then took everything back and now seriously repents, I… Then I accepted a conclusion of a single psychiatrist. I think it was a mistake. I think such decisions should be made collegially (Resp. A).

One respondent accepted patients who received the diagnosis only from one specific psychiatrist: “Recently we introduced such a rule that [initiating HRT is possible after receiving a conclusion] from my colleague-psychiatrist from the psycho-neurological dispensary, he will sign… I know him personally, so I trust him” (Resp. D). When asked about the legal basis for requiring the diagnosis, none of the respondents could give a clear answer: “In our country, everything is regulated. No matter public doctors or private, there is legislation, order of… Rospotrebnadzor… oh God, who… Roskomnadzor” (Resp. B). No such order could be found by the author. One respondent admitted: “I do not know where it is written” (Resp. D). Asked whether the informed consent model could be implemented in Russia, all the respondents gave a negative answer motivating it with concerns for their own safety:

Taking into account that in Russia in general the attitude to them [trans people] is very complicated, and even among the medical community, and taking into account our last Constitution and the general attitude towards the LGBT movement… it is very unsafe for the reputation and career of the doctor. Some side effects of therapy, God forbid, thromboembolia as a result of treatment without confirmation, only based on informed consent—I think it will be very unfortunate for the doctor … The last case of Sushkevich when they did not keep alive a premature baby. 7 years in prison (Resp. A).

Not only do the doctors want to protect themselves but also the patients. Here the argument about detransition was used again:

You need to safeguard yourself. I have patients… they underwent a commission, got evaluated… and then they came to me and said: no, they want retransition, return to their biological sex … And now they blame everyone around. [Do you want to protect yourself or the patient?] Of course. And myself, and the patient. You should think about both (Resp. D).

Understanding the barriers to obtaining the diagnosis, some endocrinologists were open to providing unofficial consultations as a harm-reduction measure:

There are cases when patients come already on therapy, they self-prescribed it and there is no certificate. And here is another story… Of course, I can help the person, but I cannot do it officially. I cannot mention it in the report, for legal reasons. But giving recommendations, so that the person does not harm himself—sure (Resp. C).

The respondents did not have clear opinions about provision of GAHT to minors, partly because five out of six worked with adults. The only pediatrician in the sample said: “I think before puberty anyway. Everyone is different, 12, 14 years old … The puberty can limit chances for a beautiful, correct transition” (Resp. F). Others believed that minors were not mature enough to make such a decision: “Now I have a young patient of 19 years old, he recently started HRT, but it is difficult. He does not know what he wants. And with adolescents it is even more difficult, I think” (Resp. A). The concept of blocking hormones also raised criticism: “It is a rather important period when the hormones are required. During the age critical for personal formation just blocking sex hormones—it will negatively affect cognitive and mental… bone density… cause somatic and mental consequences” (Resp. E).

## Discussion

Discussion of the results can be conveniently divided into two parts: experiences of receiving/providing care and opinions on the organization of care. As for experiences, the study complemented previous research showing large prevalence of self-medication among trans people around the world (Baba et al., 2022; Do et al., 2018; Haan et al., 2015; Liu et al., 2020; Maschiao et al., 2020). The reasons for choosing self-medication include the self-perceived lack of necessity, disenchantment with doctors, barriers in access (cost, age, lack of the diagnosis), and fear of mistreatment. The participants generally understood the risks and turned to doctors when they felt hormone therapy could interfere with their health conditions. However, receiving a consultation from an endocrinologist was not a sure way to avoid complications. Thus, self-medication should not be conceptualized exclusively as a forced measure caused by inaccessibility of care but as a rational choice. The informants exercised agency and weighted costs and benefits of going to a doctor or self-managing their therapy. While the surveyed endocrinologists were skeptical about self-medication, they thought it could be safe in certain cases when no concomitant diseases were present and the person had enough knowledge. Understanding the barriers leading to self-medication, some endocrinologists were willing to provide informal consultations as a harm-reduction measure.

When asked about the organization of care, a half of trans participants supported the informed consent model, while another half, as well as all the endocrinologists, believed that the current gatekeeping model was most suitable in an adverse political climate like Russian. Psychiatric assessment was claimed to protect both the patient and the doctor from the pressure on behalf of state authorities and parents. In a conservative state like Russia, this argument is well-founded. Half a year after the data were collected, Russian parliamentarians in cooperation with conservative parents’ organizations introduced a bill prohibiting legal gender recognition and gender-affirming care, claiming that medical commissions were not conducting a proper assessment and were willing to give the diagnosis to whoever paid. Some participants also believed that diagnostics could reduce the number of detransitioners. Their number in Russia is unknown. In a private talk, a psychiatrist told me about 5 detransitioners who had approached the commission in order to change their legal gender back out of approximately 1000 trans patients, giving a rate of around 0.5%. This number is probably underestimated because it included only those who had previously changed their gender marker. In a US sample, 13.2% reported ever detransitioning, with 82.5% mentioning at least one external factor (family pressure, discrimination) (Turban et al., 2021). On the other hand, a different survey found a much higher prevalence of internal motivations for detransition (Vandenbussche, 2022). Despite the rather low prevalence of regret, it is regularly used around the world to keep the gatekeeping model (Levine et al., 2022). However, this argument was not supported by the participants’ own words in this study: all the detransitioners they knew had received the diagnosis “transsexualism,” meaning that a psychiatric assessment was not a sufficient barrier to stop them from transitioning.

Providing trans healthcare to trans minors is another contested topic around the world. The previous, 7^th^ WPATH Standards of Care recommended GAHT for adolescents who had reached the age of 16 (Coleman et al., 2012). In the 8^th^ Standards, the age limit was removed (Coleman et al., 2022). In Russia, adolescents of 15 years and older can give informed consent for medical treatment without their parents’ approval. ICD-10 includes the code F64.2, which could make them eligible for GAHT. Thus, at the time of data collection, there were no legal barriers for receiving GAHT by trans minors. However, because of the hostile political climate, psychiatrists were afraid to give the diagnosis and endocrinologists were afraid to prescribe hormones. Their fears turned out to be well-founded when the 2023 bill was proposed. Its explanatory note claimed: “Currently, in Russia, there is a well-developed industry of the change of sex, including dishonest doctors, psychologists, a well-developed network of LGBT organizations and activists, other individuals. All their destructive activity are directed at adolescents and the youth.” Thus, trans minors were used as an excuse to prohibit gender-affirming care for all trans people.

The adoption of this law in July 2023 did not invalidate the findings of this study. Only a month since its coming into force it is not clear how the practices will change. There are endocrinologists that continue to prescribe GAHT to those who had their legal gender amended previously (technically speaking, provision of care to them is not considered a “change of sex”), while others found it safer to stop working with trans issues. This means that self-treatment among trans people will become even more wide-spread than before.

## Notes

1 As of this writing, Russia still uses the International Classification of Diseases, version 10 (ICD-10). Transition to ICD-11 is planned for 2024; however, there is a strong opposition on behalf of some psychiatrists and politicians calling to retain ICD-10 codes F64 and F65. The Diagnostic and Statistical Manual of Mental Disorders (DSM) is not being used in Russia.

2 The term GAHT employed throughout this text is rarely used in Russia because “affirming” has no good equivalent in Russian. Instead, trans people tend to call it “hormone-replacement therapy” (HRT).
